# The Norsesquiterpene Glycoside Ptaquiloside as a Poisonous, Carcinogenic Component of Certain Ferns

**DOI:** 10.3390/molecules27196662

**Published:** 2022-10-07

**Authors:** János Vetter

**Affiliations:** Department of Botany, University of Veterinary Medicine, Pf. 2, 1400 Budapest, Hungary; vetter.janos@univet.hu

**Keywords:** ptaquiloside, bracken species, PTA in soil, PTA in water, carcinogenic effect, animals, human, global problem

## Abstract

Previous studies related to the ptaquiloside molecule, a carcinogenic secondary metabolite known from the world of ferns, are summarised. Ptaquiloside (PTA) belongs to the group of norsesquiterpenes of the illudane type. The name illudane refers to the fungal taxa from which the first representatives of the molecular group were identified. Ptaquiloside occurs mainly in *Pteridium* fern species, although it is also known in other fern taxa. The species of the genus *Pteridium* are common, frequent invasive species on all continents, and PTA is formed in smaller or larger amounts in all organs of the affected species. The effects of PTA and of their derivatives on animals and humans are of great toxicological significance. Its basic chemical property is that the molecule can be transformed. First, with the loss of sugar moiety, ptaquilosine is formed, and then, under certain conditions, a dienone derivative (pteridienone) may arise. The latter can alkylate (through its cyclopropane groups) certain molecules, including DNA, in animal or human organisms. In this case, DNA adducts are formed, which can later have a carcinogenic effect through point mutations. The scope of the PTA is interdisciplinary in nature since, for example, molecules from plant biomass can enter the body of animals or humans in several ways (directly and indirectly). Due to its physico-chemical properties (excellent water solubility), PTA can get from the plant into the soil and then into different water layers. PTA molecules that enter the soil, but mainly water, undergo degradation (hydrolytic) processes, so it is very important to clarify the toxicological conditions of a given ecosystem and to estimate the possible risks caused by PTA. The toxicoses and diseases of the animal world (mainly for ruminant farm animals) caused by PTA are briefly described. The intake of PTA-containing plants as a feed source causes not only various syndromes but can also enter the milk (and meat) of animals. In connection with the toxicological safety of the food chain, it is important to investigate the transport of carcinogenic PTA metabolites between organisms in a reassuring manner and in detail. This is a global, interdisciplinary task. The present review aims to contribute to this.

## 1. Introduction

The primary metabolic processes of plants are the set of universal processes that serve the growth and development of plants. The processes of photosynthesis, respiration, protein, and nucleic acid metabolism can be cited here. The reactions of secondary metabolism help the survival of plants through the often very complicated relationship between the individual and the environment. The above concepts appeared for the first time in the 1870s and are generally widespread and used today. Characterization of secondary metabolic products: they are not common, they occur in specific taxonomic units, they are not energy sources, their synthesis is often tied to one stage of development, they can be stored in various ways, and in chemical terms, they have extremely diverse structures [1,2]. The secondary metabolites of the flora are closely related to the environment, as they play an important role in the abiotic and biotic environmental effects (different aspects of the ecosystem) and in the development of symbiotic relationships between species [3]. The secondary metabolic products can be grouped into five large compound categories (saccharides, phenolics, polyketides, terpenoids, and azotoids) [1].

The aim of our work is to present a summary of the new research results on a molecule known from the world of ferns, with diverse and strong biological effects, and to review the significant biological effects. This molecule is ptaquiloside, which belongs to the large group of terpenes [4].

**Figure 1:**
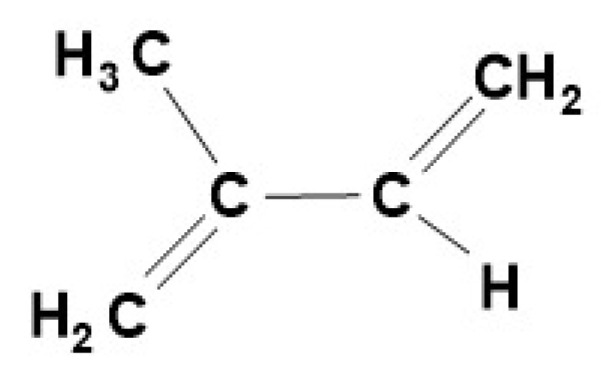
The basic unit of terpenes is the five-carbon isoprene (2-methyl-1,3-butadiene).

The basic unit of terpenes is the five-carbon isoprene (2-methyl-1,3-butadiene: Figure 1). In a more general sense, these are molecules whose structure corresponds to the formula (**C_5_H_8_**), including their oxygenated, hydrogenated, or dehydrogenated derivatives. The number of known terpenoids is in the tens of thousands, the number of new molecules described each year is in the thousands, and they are grouped based on the number of isoprene units (hemiterpenes contain one, diterpenes two, and sesquiterpenes three units, and so on) [1,2].

## 2. Discovery, Characterization, and Properties of the Molecule

**Figure 2:**
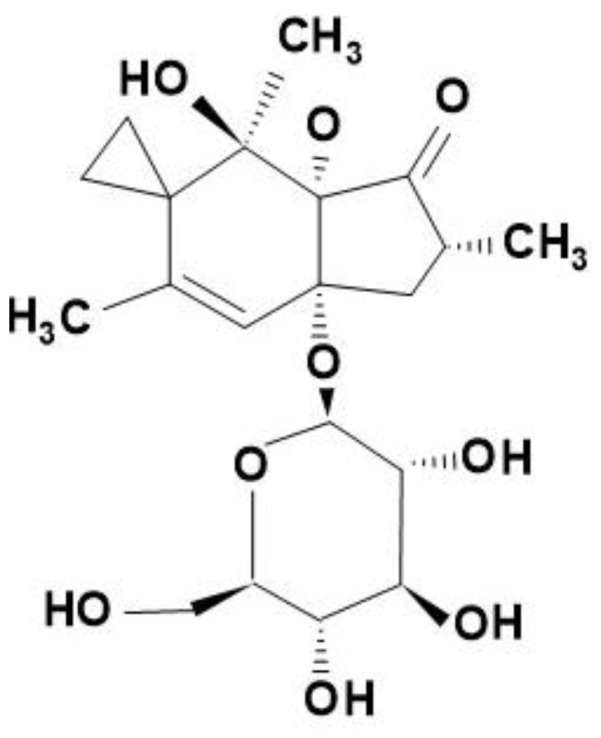
The chemical structure of the ptaquiloside (PTA) molecule.

The discovery of the ptaquiloside molecule was logically preceded by various veterinary and human medical experiences. They early drew attention to the fact that some fern species (mainly *Pteridium* species) can be the starting point of various animal diseases (toxicoses) through their consumption. The chemical analysis of the bracken fern species (*Pteridium aquilinum*) led to isolation and characterization of a poisonous new molecule [5,6,7] which was named ptaquiloside (PTA) and belongs to the norsesquiterpene glycosides of the illudane type (Figure 2).

**Figure 3:**
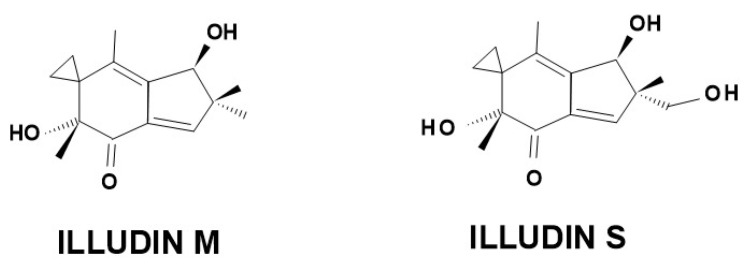
Chemical structure of the similar illudin M and illudin S compounds.

The sesquiterpenes form one of the richest groups of secondary metabolites in plant and fungal world; the **C_15_** structure allows many variations, which also causes the diversification of biological effects [8]. The name of the illudane type comes from the world of fungi, as similar molecules (illudin M and illudin S (Figure 3) are the components of some poisonous mushroom species (*Omphalotus illudens, O. olearius*). The number of illudane-type sesquiterpenes described from mushrooms is increasing due to intensive research [8].

PTA is an amorphous, colourless molecule (**C_20_H_30_O_8_**) of molecular weight 398.45; the exact chemical name is: (2*R*,3*aR*,7*S*,7*aR*)-7-hydroxy-2,5,7-trimethyl-3*a*-[(2*S*,3*R*,4*S*,5*S*,6*R*)-3,4,5-trihydroxy-6-(hydroxymethyl)oxan-2-yl]oxyspiro[3,7*a*-dihydro-2*H*-indene-6,1′-cyclopropane]-1-one. Characteristics: melting point is 85–89 °C, UV absorption maxima at 214, 220 nm [9]. PTA is readily soluble in water and is stable at room temperature for more than a week and at lower temperatures for several months [10].

## 3. Biosynthesis

**Figure 4:**
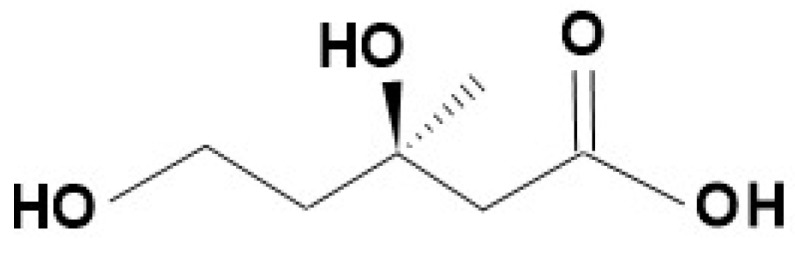
The mevalonic acid (3,5-dihydroxy-3-methylvaleric acid), an important member of the terpene biosynthesis.

The biosynthesis of terpenoids—linked to the primary metabolism of plants—can be summarized below. The first step is the creation of the isoprene backbone (IPP: isopentenyl pyrophosphate), which is produced mainly by the mevalonic acid route and partially by the glyceraldehyde phosphate/red vinyl acid route). Mevalonic acid (3,5-dihydroxy-3-methylvaleric acid: Figure 4) is produced from 2 molecules of acetyl-CoA and condensed with a third acetyl-coenzyme A through aceto-acetyl-coenzyme A, to 3-hydroxy-3-methyl-glutaryl CoA, followed by its reduction. This is followed by a two-step phosphorylation, with the use of 2 ATP; then the decarboxylation of the diphosphate derivative and the cleavage of inorganic P occur, thus producing the IPP [11].

In the second stage, the **C_5_** units (IPP and its isomer DMAPP/dimethylallyl pyrophosphate/) condense and form three types of prenyl-diphosphates (**C_10_**: geranyl-PP, **C_15_:** farnesyl-PP and **C_20_:** geranyl-geranyl-PP). Later, sesquiterpenes are formed from farnesyl-PP, and then in further reactions, a variety of molecular transformations (oxidation, reduction, conjugations, etc.) can occur (including the very frequent formation of glycoside derivatives), which can lead to hundreds of terpene derivatives. Most of the sesquiterpene derivatives of fungi are found among the basidiomycetous fungi [12]. Sesquiterpenes of fungal origin are also formed mainly via the mevalonic acid pathway. The reactions for the formation of mevalonic acid require several enzymes (AACT,/Acetoacetyl-coenzyme A thiolase/, HMGS/Hydroxymethylglutaryl-CoA synthase/, and others). The discovery of illudins in the 1950s initiated research into this sesquiterpene group, which aims not only at the isolation of new and new molecules, their structural research, and the investigation of their biological effects, but also at the implementation of in vitro synthesis [8,13].

## 4. Decomposition (Transformation) of the Molecule

**Figure 5:**
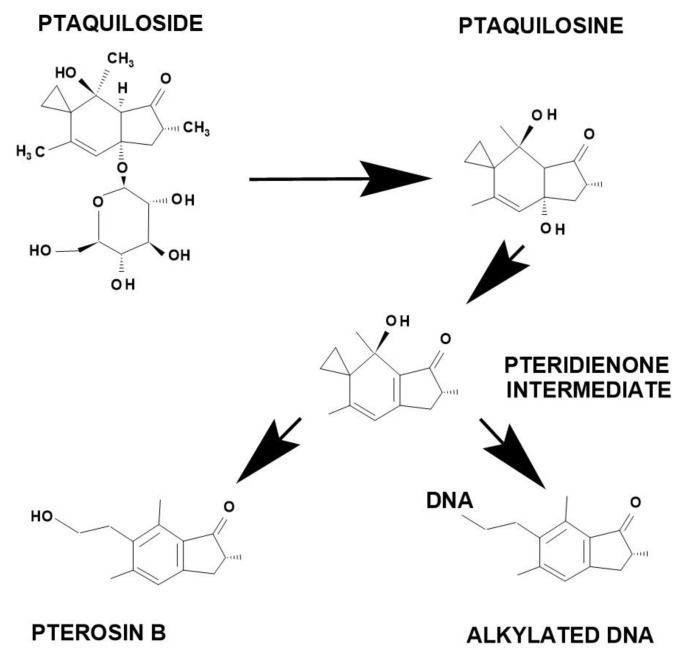
The possible metabolic ways of ptaquiloside (PTA) transformations. From the dienone intermediate (pteridienone) the non-toxic pterosin B can be produced, or the DNA molecule can be alkylated, which can lead to carcinogenesis through mutations.

PTA can go through decomposition processes according to environmental conditions (mainly pH, temperature). As a result, various metabolites are produced, the effects of which are decisive from a biological point of view. All questions of decomposition (hydrolysis) have become the focus of interest and investigations, especially those related to toxic (carcinogenic) molecules and their effects [14]. The possible pathways of PTA metabolism are shown in Figure 5. The molecule is mainly degradable in acidic and alkaline media. Under alkaline conditions, the glucose molecule breaks down (with an enzymatic reaction) and an aglycon-type molecule called ptaquilosin is produced (Figure 5). which then forms a dienone intermediate (pteridienone) (Figure 5). After that, a different route is possible, with the dienone absorbing water, the aromatic pterosin B (PTB) molecule is formed (Figure 5), which is inactive and does not have a significant biological effect. The second possibility is that the pteridienone can covalently bind to DNA molecules and thus can produce an alkylated DNA by-product that did not exist before. These alkylated DNA by-products are the main actors in the carcinogenic effect of PTA, and there has long been no doubt that these molecules play a decisive role in carcinogenesis, since by changing the genetic code they create mutations, aberrations and thus cancers. The reaction between pteridienone and DNA forms adducts through N-3 of adenine or N-7 of guanine [15]. DNA alkylation caused by pteridienone seems to be the molecular basis of bracken fern-induced carcinogenesis. If the PTA molecule is degraded to pterosin B (PTB), the described problems do not occur. Therefore, the tests (see later) that investigate the fate of molecules entering the soil and/or water (water base) from the plant are very important.

**Figure 6:**
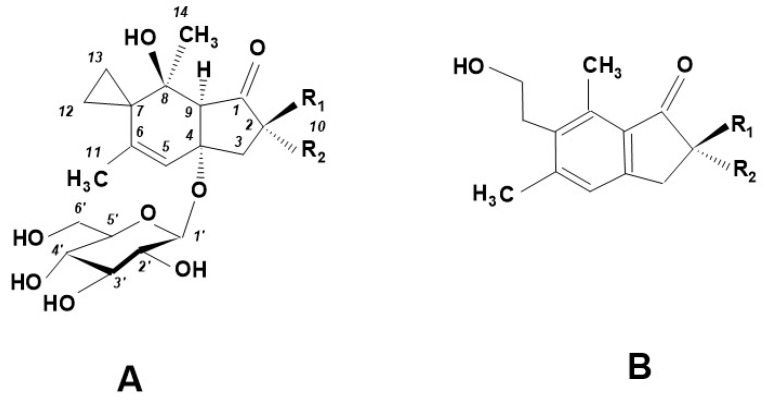
The basic structure of ptaquiloside and its derivatives (PTA, CAU and PTE: **A**), and of the pterosins (pterosin G, pterosin A and pterosin B) that can be formed from them (**B**) (based partly on [19]).

Nowadays, several PTA derivative molecules have been isolated and identified from other fern species: for example, iso-ptaquiloside (which is an isomer of PTA), caudatoside (CAU) from *Pteridium caudatum* [16]; ptaquloside Z also from *P. caudatum* [17]; PTE (ptesculentoside, detected from *P. esculentum* [18]. The corresponding pterosins are produced from each of the listed derivatives during degradation. The chemical relationship between PTA derivatives and the pterosins produced from them is shown in Figure 6 and in Table 1 [19]. Table 1 indicates clearly that PTE is transformed to Pterosin G, CAU to Pterosin A and (most importantly for us) PTA to Pterosin B. PTA and/or its derivatives have been detected in more than 50 fern species. The PTA, CAU and PTE molecules have the identical structural framework, the differences between these molecules are in active hydrogen bridge promoters–hydroxy groups. These groups facilitate binding processes, for example to amino acids, water molecules or N-containing components. The PTA molecules (Figure 2) contain the electrophilic cyclopropane functional group, which is responsible for alkylation of DNA, i.e., for the later genetic (carcinogenic) effect. 

Other derivative molecules (for example, pteridanoside) contain a cyclobutene group instead of a cyclopropane group. The pteridanoside (from bracken fern) is sixteen times less toxic than the ptaquiloside. Compounds without the cyclopropane or cyclobutene groups are not considered as carcinogenic.

The specific R_1_ and R_2_ groups and the molecular forms of the compounds are shown in Table 1.

PTA analogues (derivatives) may play a role in the main biological effect of the molecular group (carcinogenicity), but even today most data are almost exclusively focused on PTA, so this molecule is included in most works as the only carcinogenic substance. In Kisielius’s dissertation [20], he also provides a chronological overview of the recognition of the problems and the time when each derivative was detected. The data indicate that the poisonous nature of *Pteridium* ferns on farm animals was indicated as early as 1893, while the importance of CAU, PTE was revealed in the last few years [19]. Another issue is that, according to recent observations, PTE and CAU derivatives are more prone to leaching from the plant with precipitation than PTA [20].

## 5. The Most Important PTA Carrier Plants, the *Pteridium* Species

The molecule in question (and its derivatives) belong mainly to the genus *Pteridium* (the family Dennstaedtiaceae, phylum Pteridophyta). The species of the genus have a very wide ecological amplitude, occurring on all continents except Antarctica. It is likely that they were very widespread in the Oligocene age (about 23–34 million years ago). According to today’s surveys, their coverage, which can be measured on agricultural production sites, is very large; the species *P. aquilinum*, for example, spreads over nearly 1.7 million hectares in the United Kingdom, which is 7.3% of British agricultural land [20].

An overview of the species in the genus is presented in Table 2, based on work of Cacador, 2014 [21]. The most important species is the bracken fern (*P. aquilinum* (L.) Kuhn, of which 11 subspecies are known. The subspecies aquilinum is native in Europe. *P. aquilinum* is a very aggressive, invasive plant, which can also survive extreme conditions. It is a perennial fern with a branching rhizome, which produces its erect leaves with long petioles that grow up to 2 meters. The leaves are botanically two- to four-fold, the backs are slightly fluffy, the spore holders are located on the edge of the leaf wings. The colour of the leaves starts to change at the end of summer, and they are usually killed by the first frosts. Development of the plant occurs from the spores but the bioproduction of the plant is possible from the strong rhizome. The rhizome is an organ with a diameter of 2–2.5 cm, which is mainly used to store reserve nutrients. The number of spores produced by an individual bracken fern can be up to 30 million, and its spores retain their viability for almost 10 years [22].

Under appropriate ecological conditions (pH: 5.5–7.5, temperature 15–30 °C), the emergence of young plants takes 6–7 weeks after spore scattering. The bracken fern occurs in large numbers, often in dense stands; in Europe it can be part of different forest patches (deciduous and conifer associations), and is found partly in areas with shrubs, hilly areas or even in plains. In Hungary, we can also classify it among the common “weeds”, the largest populations of which can be found in Western Transdanubia and in Nyirség (Eastern Hungary) [23]. *Pteridium aquilinum* (and other P. species) are particularly suited to populating (conquering) new areas because it has more competitive advantages compared to other plants [20]. These include the successful struggle for light (it can strongly cover other species) and successful access to water (through the rhizome system). After natural disasters (such as fire), it continues its life quite successfully and supplants competing plants.

One of its competitive advantages lies in its chemical composition, as it contains various repellents and/or toxic substances that act on herbivores. It can hinder the germination, growth, and bioproduction of some plants (obviously through allelopathic molecules and/or effects). Its chemical composition contains a wide variety of molecules that affect other organisms, as it contains antithiamine factors, cyanogenic glycosides, and the illudane-type sesquiterpene glycosides. Many data in the literature indicate that the complex biological effect of this rich group of compounds creates the “deterrent effects” exerted on fungal pathogens, insects, or even ruminant animals [20].

Ptaquiloside molecules has been found not only in *Pteridium* species, but in *Pteris cretica*, *Cheilanthus myriophyla* (from Pteridaceae family), in *Dennstaedtia scabra*, *Histiopteris incisa* (from family Dennstaedtiaceae), or in *Cibotium barometz* (fam. Cybotiaceae), in *Onychium cryptogrammoides*, *O. tenuifrons* (from Pteridaceae family), in *Dryopteris cochleata* (fam. Dryopteridaceae), in *Hypodematium crenatum* (fam. Hypodematiaceae) [24,25,26]. 

## 6. PTA Contents and Distribution in Plants

The concentrations of PTA measured in plants can be reviewed in Table 3 based on data from the literature. Samples of different *Pteridium* species (mainly *P*. *aquilinum*) from very different geographical locations were analysed. In the table, the data are presented in mg/g units, or converted to such units.

Unfortunately, for the data from several sources, it is not clear whether measurements applied to the unit of fresh weight or dry weight of the plant. The PTA concentrations are found in a wide interval, which is also explained by the understandable differences of many factors. Some aspects: The samples of *P. arachnoideum* from Brazil [34] are distributed in a very narrow interval: between 2.49 and 2.75 mg/g. Elsewhere (in Bolivia, for example), the PTA concentration interval of the plants of the different growing areas is very wide (ranges between 1.45 and 14.7 mg/g). The same can be seen in the data series from southern Italy (values between 2 and 780 mg/g). Other data indicate that the developmental state of the plants can fundamentally influence the measured PTA level: the mature foliage contains only about a sixth of the PTA level of the sprout samples (Table 3). Research of Zaccone et al. found in southern Italy [33] a positive and significant correlation between the total P content of the plant and the PTA level: the higher the phosphorus content, the higher the PTA level. The phenomenon can perhaps also be explained by the fact that the biosynthesis of the sesquiterpenoid requires pyrophosphates, so the biosynthesis is logically dependent on phosphorus.

### PTA Levels in Plant Organs

Rhizomes generally have lower concentrations (less than 1.2 mg/g) during leaf development than are found later in mature fronds. Storage rhizomes contain more PTA than those carrying the shoot system [10]. The PTA level of the root system developing from the rhizome is very low (0.005–0.230 mg/g). Rasmussen’s earlier test series can be a guideline [38], where the average PTA level is 3.8 mg/g (min.: 0.28, max.: 13.3). According to Rasmussen’s latest data set [39], the foliage is always the source of the most PTA; the PTA concentration is between 1 and 50 mg/g in the spring, the concentrations in the rhizome are negligible. The PTA content of the spores is very low, mostly less than 20 µg/g, i.e., 0.002 mg/g. Pursuant to other objectives, the PTA and PTB content of various products from the food industry and traditional medicine were examined [39]. It is reassuring that Rasmussen’s data (see previous literature) from the examination of a wide variety of homeopathic pills and solutions did not contain detectable amounts of PTA. The PTA levels found in the Far Eastern preparations of dried croziers were low (40–660 µg/g), and PTA molecules were only detected in the rhizome preparations. It should be noted, of course, that in many countries of the world, plants of the *Pteridium* genus cannot be included in edible plants or in food products.

## 7. PTA in Soil and in Water

The first, very surprising data that PTA molecules can be detected in the soil around *Pteridium* species are already several decades old. Since then, this phenomenon has been confirmed many times over, so today we have an acceptable picture of the plant-soil relationships. As we mentioned earlier, the ferns (especially the *Pteridium* species) can contain significant amounts of PTA (and their derivatives), but at the same time, the biomass production is very significant, or in other words, up to several kg per hectare covered by the plant [30]. Earlier, we showed that PTA is very water-soluble, so it can dissolve either from living plants through precipitation, or later, in autumn and winter, from dead plant material and become a constituent of groundwater. The PTA molecule is not strongly bound to the soil particles, so it always remains a component of the aqueous phase. In a soil layer of 90 cm a variable PTA level was found up to a concentration of 7 µg/L [40]; the PTA tends to be washed by precipitation into the different water layers of the soil. Investigations in Denmark, the UK and Ireland have confirmed that the leaching of PTA into different water bodies (groundwater, surface water) is an existing phenomenon. PTA levels of 0.6 µg/L (in Irish groundwater), 0.09 µg/L (in Denmark) were measured [40], while concentration of up to 2.2 µg/L formed in surface waters because of rains. 

We may summarize what we have now established about the PTA-carrying plants, their soil environment, and the possibility of it leaching into water. The plant cover is very large, it is the fifth-rated weed plant on Earth (see earlier) and it potentially produces a lot of toxic metabolites. Let us make a theoretical, hypothetical, but data-based calculation. In the UK, the area covered by bracken fern is estimated to be 1.7 million hectares; if the plant weight contains only 1 kg of PTA per hectare, the total PTA weight in question is 1700 tons. Hence, the fundamental question is what amount of PTA gets (may get) into the waters. In general: what factors affect hydrolysis and, if necessary, what water purification procedures can restore the water quality? 

According to experiments carried out on soil types, the degradation rate is between 0.061–0.090 × 10^−2^ mg kg^−1^ day^−1^. In the soil type with lower carbon- and higher sand content, the rate of degradation was higher than in the soil type with higher carbon and lower sand content. This fact confirms the finding of Rasmussen et al. [14] that the affinity coefficient of PTA depends linearly and positively on the soil clay and organic matter content. The smaller the amount of clay and organic matter, the less PTA binds, i.e., the faster it decomposes. The role of the soil’s microbial community in decomposition is also clear today, as hardly any decomposition occurs in sterile soil. The half-life of PTA molecules in soil is between 8 and 180 hours and mainly depends on the soil parameters [41]. Regarding the rate of PTA leaching from plants, according to the results of Garcia-Jorgensen [42], 0.2% of the PTA present in the above-ground biomass is washed into the soil, per mm of precipitation. The amount entering the soil is inversely proportional to the depth of the soil, and the PTA concentration of the soil solution depends on the PTA level of the plant biomass. 

### Getting PTA into the Water

In recent years, several fundamental scientific works have been published, mainly in places where previous investigations have already laid the foundation for this topic (U.K., Denmark, Ireland). Skrbic and co-workers [40] collected and analysed Danish, Swedish and Spanish water samples (PTA, CAU, PTE and the corresponding hydrolysis products were measured). The deep springs (40–100 m) contained neither illudane glycosides nor pterosins. The seven shallow springs contained at least one illudane glycoside and pterosins according to the following average concentrations: PTA: 0.27 µg L^−1^; CAU: 0.75 µg L^−1^; PTB: 0.05 µg L^−1^; PTA: 0.03 µg L^−1^ and PTG: 0.28 µg L^−1^.

According to previous, sporadic data, PTA in different water bodies in spring water in Ireland fluctuates between 0 and 0.09 µg L^−1^ in groundwater samples close to the surface, in Denmark its content is 2.2 µg L^−1^. The tests were mainly aimed at the Danish/southern Scandinavian regions, because due to the climatic conditions there (intense precipitation in the colder periods, and significant bracken cover), it is likely that PTA is washed into the soil. Compared to the waters of deep springs that do not contain illudanes at all, the polluted, shallow springs have a neutral pH and lower electrical conductivity.

Irish tests [43] found PTA in all drinking water samples (0.67 µg L^−1^; 0.57 µg L^−1^ and 0.01 µg L^−1^). It seems logical to assume that the PTA level of the plant proportionally affects the amount washed into the water layers. However, the complexity of the situation is shown by the fact that from the plant with a PTA concentration of 27.6 µg/g^−1^, only 0.01 µg/L^−1^ was measured in the soil solution under the bracken fern, while 0.67 µg/L^−1^ was measured in the water under ferns with a PTA level of 3.33 µg/g^−1^.

In Denmark in 2021, in relation to waterworks, they investigated which methods are suitable for the degradation and removal of PTA (300 µg/L, a very high concentration) [44]. Despite the extremely high PTA levels, all types of sand filters successfully removed the material. Following the breakdown of PTA, degradation products (Pterosin A, B) were detected. 

Filter sand with the best cleaning effect was characterised by a high amount of deposited iron and manganese oxides (i.e., surface areas are high). Fast degradation of PTA and CAU was observed, and the pollutants were hydrolysed completely within the first half hour; the “cleaning” effects of other types of filter sands were something lower.

## 8. Biological Effects

The biological effect of PTA can arise from its direct entry into animal (human) organisms from the carrier plants, i.e., through nutrition [45]. In other cases, the introduction of PTA is preceded by the transformation of the molecule. Cattle that eat bracken excrete 8–10% of the molecule into milk, which can then be used for human consumption. An indirect possibility is provided by the fact that a small part of the PTA in the plant mass can be washed out, transferred into different water layers or even into the sources that serve as the basis of drinking water.

### 8.1. Effects on Animals

#### 8.1.1. Ruminants (Cattle, Sheep)

The chronic form of the disease, Bovine Enzootic Haemature (BEH), develops mainly in the case of cattle. It can be characterised by different bleedings (haemorrhages), later tumours (mainly in the urinary bladder) and by bloody urine. The BEH syndrome occurs in the most diverse geographical areas of the world, where the role of *Pteridium* ferns in the fodder source of animals is decisive [5,10,24,45].

Unambiguous correlation was documented between BEH and PTA content of bracken by epidemiological studies. The clinical symptoms are losses of condition, dyspnoea and noisy breathing, salivation, halitosis, haemature. Histopathologically: epithelian and mesenchymal tumours were found in the urinary bladder. The haematological status of the affected animals can be characterised by low haemoglobin content (6.88 g/dL), packed cell volume, total erythrocyte count: 4.43 ± 0.4 × 10^6^/µL. All animals had hypoglycaemia, hypocalcaemia and hypophosphataemia [46].

In addition to the tumour of the urinary bladder, additional problems occur in the body of sick animals: metastasis of adenocarcinoma in lung, liver, even in the heart of some animals [47]. During the consecutive consumption of PTA-containing plants (after 30 days), higher creatinine, urea, uric acid, and phosphatase activity are characteristic in animals. After 45 days, epithelia cells and calcium oxalate crystals also appear in the urine. The veterinary literature has already reported data on PTA concentrations in the animal body [48]. Contents of PTA, PTB and pterosin G were measured. PTA content of plasma of 0.97 mg/mL, and 1.30 µg/mL of ptesculentoside was found, but these values depleted (to <10% of these values) within 24 hours after bracken consumption. Fifteen days after taking the bracken, 0.42 µg/g and 0.32 µ/g of ptesculentoside were present was in skeletal muscle and liver, respectively. The fate of the norsesquiterpene glycosides remaining in the animal organisms requires further studies.

Aranha and co-workers [49] followed the fate of PTA administered to cattle (intravenous administration of PTA). The large fraction of PTA was converted to PTB, both PTA and PTB were excreted in urine (up to 40% of the dose!). It is noteworthy that the oral administration of PTA (via extract or dried form of fern) did not cause the appearance of PTA molecules. All this indicates that deglycosolidation takes place in the rumen, and PTB can be detected both in the plasma and in the urine. In case of treatment of 7 days of PTA (2 mg PTA/kg), the experimental animals developed preneoplastic lesions in urinary bladder.

#### 8.1.2. Sheep

Although sheep are generally less sensitive to toxic factors than cattle, BEH can also occur in sheep, and there have even been mass poisonings (in Australia, 120 animals died out of 450). Other problems: a progressive retinal degeneration (PRD or bright blindness) was developed by sheep in some areas of the UK in the middle of 1960s; later, it was described in New Zealand and in Australia too. Long-term (months, rather years) penetration of PTA causes this special symptom. Affected sheep may be practically blind, and pupils respond poorly to light [44]. Animals show a degeneration of neuroepithelium of the retina, and they may have many other lesions (bone marrow suppression, haemorrhage, neoplasia of urinary tract). The PTA molecules have a definitive role in PRD, since it can be reproduced experimentally both with powdered bracken plants and with purified PTA [5].

### 8.2. Effects on the Human Organism 

The historical background of the human consumption of the fern is not easy to outline. It is likely that over-consumption would only have taken place in extraordinary situations (famines, war, floods), using either ground rhizomes or with parts of the shoot system. In the case of the Ice Man, it is certain that our 5,300-year-old ancestor consumed the plant [50]. Interestingly, ferns have been used for various purposes throughout history (as food, medicine, ornaments, etc.) The number of edible (or considered as edible) fern species in China is 144 [51]; the ostrich, bracken and royal ferns are the most popular edible fern species.

*Pteridium aquilinum* served as human food during the First World War in Scotland and was collected and consumed in North America (USA, Canada), Russia, China, and Japan. More recently, in some places, the young fern has appeared on the menus of gourmets as a gastronomic specialty. Before consumption, it is usually boiled in water (salt water), while various chemicals are also used to reduce the carcinogenic effect [5].

In some countries (Japan, Brazil, Venezuela [5]) a close connection was found between consumption of bracken fern and cancers of upper alimentary tract [52]. The frequency of oesophageal and gastric cancer in these countries has increased several times compared to non-consumers of the fern.

The fundamental question is how the active ingredients of the fern (mainly PTA and its derivatives) can enter the human food chain indirectly? In Wales, for example, the risk of cancer increased by 2.34 times for people who, from childhood, regularly consumed milk from bracken-infested areas [5]. A similar increase in risk occurred in Costa Rica. Alonso-Amelot’s working group showed [48,53] that 8.6% of consumed PTA can be secreted in the milk of cattle.

The milk element of the food chain can have other significance. Virgilo’s working group [54] collected milk samples from different sheep and goat herds in Italy. The animals grazed on plant associated with a predominance of bracken; however, in control areas bracken did not occur on the pasture. The PTA content was easily measured in the milk samples collected between May and September of the animals living in the fern areas: the data ranged from 0 to 1.63 ng/mL in the sheep and 0.57 to 3.14 ng/mL in the milk collected from the goats. The mixed milk of the animals in the control areas did not contain PTA.

The main conclusion of the test series is that the milk of otherwise healthy (showing no symptoms) livestock living and feeding in the given area can cause toxicological problems. All of this is an undervalued, global issue of food safety.

Possible targets of PTA transfer (transport) are other organs and tissues of the animal body. In connection with the food chain, the possibility of contamination of meat may legitimately arise. Although some studies [49,55] already suggest that PTA and its derivatives can enter the different organs of animals, indicating the possibility of contaminated meat (meat products), further studies are needed.

### 8.3. Spores

Spores of bracken can be potentially risky for the local people such as forestry workers of bracken infested areas. On an eagle fern plant, for example, during the spore maturation period (from July to September), several grams of spores can be produced [56]. The large plant stock, therefore, also represents a potential toxicological risk. In a study, 53 mice within a group of 98 animals dosed with spores developed tumours. In the upper gastrointestinal tract of spore administered mice, formation of DNA adducts was observed [45]. PTA content of bracken spores was quantitatively determined in a collection of spores, originating from Britain [56]. PTA was found in all samples and the highest content reached 29 µg/L. The risk of poisoning with spores is significantly lower compared to milk or drinking water.

## 9. Control of PTA Carrier Ferns

The International Agency for Research on Cancer (IARC) classified ptaquiloside as a chemical carcinogen in class 2B, where compounds are probably carcinogenic to humans [45].

Due to the previously outlined biological effects of PTA, its interpretation and registration as a carcinogenic molecule, the control of the spread of carrier plants and the suppression of plants in each area is an important issue. Limitation of Pteridium species is also possible with some traditional agricultural methods [14]. The operations of cutting of the aerial part or deep ploughing carried out for years can lead to partial success. Studies indicate that the first cut should be carried out in early spring, after which the shoot system will roughly regenerate from the rhizome, so that the following second cut effectively consumes the nutrient reserves of the rhizome. The treatment carried out in this way for five years reduced the dry matter production of the plant per area unit by 60% [14].

Chemical options for weed control include the use of systemic herbicides. Thus, Glyphosate, Asulam, Asulax have been used, with low contents of surfactants. The aim of these treatments was to transport the herbicide molecules to the buds of fern rhizomes. Several adverse effects can be experienced in connection with such experiments; moreover, the chemicals taken up by the shoot system do not reach the rhizomes, so over time the original metabolic situation is restored. It was also found [45] that the agents applied to the bracken fern clearly destroy other fern species, which changes the biodiversity of the ecosystem. Glyphosate and methyluron also affect many other plants, causing further changes in biodiversity. In addition, the use of certain herbicides, especially glyphosate, is only permitted for a certain period [57,58].

In principle, the spread and production of the bracken fern can be limited by different biological methods and by means of their pests. For example, the larvae of the butterfly species *Conservula consigna* consume the eagle fern or the fungus species *Ascochyta pteris*, i.e., they can damage the plant. Despite these efforts, biological control experiments have not been successful [45].

## 10. Conclusions

The topic of our review is the norsesquiterpene ptaquiloside (PTA), a secondary plant metabolite with a strong biological effect. While the negative biological effect of its carrier plants (mainly the species of Pteridium fern genus) has been suspected since 1893, the details and the mechanism of the effect were revealed much later, at the end of the twentieth century. Why is PTA information of “global” importance? Partly because of the specific botanical background (huge plant cover and biomass on five continents, the affected ferns are invasive, very vigorous plants, and all their organs contain PTA), partly because the water-soluble PTA molecules can be transformed in several ways. Through precipitation, the molecule can wash into the soil, from where—depending on the geological conditions of the area—it can reach the various water layers, even the drinking water bodies. A significant part of the PTA content degrades (can degrade), and it can be removed by cleaning (filtering) methods. Therefore, it is very important to monitor the PTA content of the water base and, if necessary, filter it with a cleaning method. The direct consumption of the plant can be carcinogenic in the long term and consumption should be avoided as much as possible. The PTA level can be reduced by various methods (e.g., chemicals). Indirect PTA ingestion is also possible since, e.g., cattle (sheep, goats) transfer (excrete) 8–10% of the PTA consumed by the plant into their milk. Prolonged consumption of such milk has been proven to multiply the risk of certain types of human cancer (in oesophagus, stomach, etc.).

PTA intake (mainly in our ruminant farm animals) is a global problem, partly because of the effects on the animal itself, and partly because of human consumption of animal products. One solution to the problem is to minimize the affected plants by weed eradication, which is very difficult due to the extreme vitality of the plants. Dangerous herbicides would be needed, which are less and less permitted for environmental reasons.

For the future, intensive research is needed in the following areas:

- Reducing the population of PTA-containing plants with new, environmentally friendly procedures.
- Monitoring the PTA content of soil and water bases in the affected areas and breaking down the measured pollutant PTA content.
- Continuous monitoring of the milk quality of the affected livestock (ruminants).
- Detection of PTA (and its derivatives) still entering the animal and/or human body, and reduction (avoidance) of possible genetic damage (DNA alkylation).

Since PTA is one of the relatively small numbers of plant-derived carcinogenic compounds, it is worthwhile to carry out larger, interdisciplinary research projects in this field.

## Figures and Tables

**Table 1:** Chemical structure of PTA derivatives (based on [19]). See also Figure 6.

	Ptesculentoside (PTE)	Caudatoside (CAU)	Ptaquiloside (PTA)	Pterosin G	Pterosin A	Pterosin B
R_1_	**H**	**CH_3_**	**H**	**H**	**CH_3_**	**H**
R_2_	**CH_2_OH**	**CH_2_OH**	**CH_3_**	**CH_2_OH**	**CH_2_OH**	**CH_3_**
Molecular formula	**C_20_H_30_O_9_**	**C_21_H_32_O_9_**	**C_30_H_30_O_8_**	**C_14_H_18_O_8_**	**C_15_H_20_O_3_**	**C_14_H_18_O_2_**

**Table 2:** The most important *Pteridium* species and subspecies of the world (after [21]).

Species	Subspecies	Occurrence
*Pteridium aquilinum* (L.) Kuhn	aquilinum	Europe
	pinetorum	Europe
	japonicum	Asia
	wightianum	India, Southeast Asia and Northern Australia
	decompositum	Hawaii
	capense	Sub-Saharan Africa
	centrali-africanum	Sub-Saharan Africa
	latiusculum	North America
	pubescens	North America
	pseudocaudatum	Eastern North America
	feei	Central America
*P. esculentum* (G. Forst.) Cockayne		South Hemisphere (Australia)
*P. arachnoideum* (Kaulf.) Maxon		South Hemisphere (South America)
*P. caudatum* (L.) Maxon		Southern and Central America
*P. semihastatum* (Wall. ex J. Agardh) S.B. Andrews		South-East Asia and Northern Australia

**Table 3:** PTA contents in *Pteridium* ferns.

Species	Plant Sample	Geographical Origin	PTA Content (mg/g)	Literature
*P. aquilinum* var. arachnoideum	whole plant	Bolivia (Rio Negro, La Cueva, La Mision, San Andres, Lampezaro	1.45–14.7	[27]
*P. aquilinum*	Mature fronds	Venezuela	1.78–1.96	[28]
*P. aquilinum*	Mature fronds	Denmark	0.21–2.14	[29]
*P. aquilinum*	Mature fronds	Scotland	0.09–2.45	[30]
*P. aquilinum*	Mature fronds	India	0.035	[31]
*P. aquilinum*	Sprouts	Venezuela	1.88–2.34	[29]
*P. aquilinum*	Sprouts	Sao Miguel Island, Azores, Portugal	3.79–6.53	[32]
*P. aquilinum*		Denmark (different locations)	1.3, 0.005 mg/g dry weight	[19]
*P. aquilinum*		Sweden	1.5; 0.033	[19]
*P. aquilinum*		U.K. (different locations)	0.042; 0.028; 0.21	[19]
*P. aquilinum*		Tanzania	0.788; 0.034	[19]
*P. aquilinum*		U.S.A.	0.057; 0.077	[19]
*P. aquilinum* ssp. *aquilinum*	Populations of different density	South Italy	2–780	[33]
	Brown, dry plants	South Italy	0.015–6.0	[33]
*P. arachnoideum*	Mature green fronds	Brazil (Conselheiro, Lafaiete, Esmeraldas, Ouro Branco, Minas Gerais, Canela, Novo Petropolis) average of these locations	2.49–2.75 2.65 ± 0.47	[34]
	Sprouts	Brazil (Conselheiro, Lafaiete, Esmeraldas, Ouro Branco, Minas Gerais, Canela, Novo Petropolis) average of these locations	12.41–18.81 16.14 ± 3.88	[34]
*P. arachnoideum*	Fresh, young leaves	Ecuador	0.59	[35]
*P. arachnoideum*	Dry, young leaves	Ecuador	2.14	[35]
	Fresh, mature fronds	Ecuador	0.56	[35]
	Dry mature fronds	Ecuador	1.82	[35]
*P. esculentum*	Mature fronds	New Zealand	2.30	[36]
*P. esculentum*		Australia	0.79	[30]
*P. species*		Australia	0–12.94	[37]

## Data Availability

Not applicable.
